# The levonorgestrel intrauterine device in Australia: analysis of prescribing data 2008–2012

**DOI:** 10.1186/s12905-018-0680-3

**Published:** 2018-11-27

**Authors:** Amie L. Bingham, Cameryn C. Garrett, Christine Bayly, Anne M. Kavanagh, Louise A. Keogh, Rebecca J. Bentley, Jane S. Hocking

**Affiliations:** 1 Centre for Health Equity, Melbourne School of Population & Global Health, University of Melbourne, 207 Bouverie Street, Carlton, 3010 Australia; 2 The Royal Women’s Hospital, 20 Flemington Road, Parkville, Melbourne, VIC Australia; 3 Centre for Epidemiology & Biostatistics, Melbourne School of Population & Global Health, University of Melbourne, 207 Bouverie Street, Carlton, Melbourne, 3010 Australia

## Abstract

**Background:**

Unplanned pregnancy is a significant problem in Australia. Local data pertaining to use of the levonorgestrel-releasing intra-uterine device (LNG-IUD), and associated factors are limited. The aim of this analysis was to calculate prescribing rates of the LNG-IUD in Australia, including trends in prescribing and associations with socio-demographic factors, in order to increase understanding regarding potential use.

**Methods:**

We examined prescriptions for the LNG-IUD recorded in the national Pharmaceutical Benefits Scheme (PBS) from 2008 to 2012. Prescribing trends were examined according to patient age, remoteness of residential location, and proximity to relevant specialist health services. Associations between these factors and prescription rates were examined using poisson regression. Analyses were stratified by 5-year age-groups.

**Results:**

Age-adjusted prescription rates rose from 11.50 per 1000 women aged 15–49 (95% CI: 11.41–11.59) in 2008 to 15.95 (95% CI:15.85–16.01) in 2012. Prescription rates increased most among 15–19-year-olds but remain very low at 2.76 per 1000 women (95% CI: 2.52–3.01). Absolute increases in prescriptions were greatest among 40–44-year-olds, rising from 16.73 per 1000 women in 2008 (95% CI: 16.12–17.34) to 23.77 in 2012 (95% CI: 22.58–24.29). Rates increased significantly within all geographical locations (*p* < 0.01). Non-metropolitan location was significantly associated with increased prescribing rates, the association diminishing with increasing age groups.

**Conclusions:**

Prescription of LNG-IUD in Australia is very low, especially among young women and those in major cities. Service providers and young women may benefit from targeted education outlining use of the LNG-IUD, strengthened training and referral pathways. Disparities in prescription according to location require further investigation.

## Introduction

The levonorgestrel-releasing intrauterine device (LNG-IUD) is a highly effective long-acting reversible contraceptive (LARC). While shorter-acting contraceptives such as the oral contraceptive pill require high levels of patient compliance to maintain effectiveness, the LNG-IUD is effective for up to 5 years, with a first-year failure rate similar to that of sterilisation (0.2%) [1]. It is cost-effective and suitable for the vast majority of women [1, 2]. While pain and changing bleeding patterns are common causes of discontinuation, reduced bleeding and amenorrhoea are considered benefits by many: the LNG-IUD can be used to treat heavy menstrual bleeding, and has a role in the prevention and treatment of endometrial hyperplasia [1, 3]. In Australia, insertion of LNG-IUDs may be undertaken by specialist obstetricians/gynaecologists, General Practitioners and appropriately trained nurses, in either clinics or hospitals [4].

Use of the LNG-IUD and other IUDs remains low in Australia: limited data suggest that IUDs are used by between 3.2 and 6.1% of contracepting women, and by as few as 1.7% of women of reproductive age in Australia and New Zealand [5–7]. Other countries considered to have low uptake at a comparable time period include Germany (5%) and the United States (5.3%) [7]. Low uptake in Australia persists despite the LNG-IUD being listed in 2003 on the Pharmaceutical Benefits Scheme (PBS) which heavily subsidises its cost for Medicare-eligible residents of Australia.

Given its safety and efficacy, there have been calls for efforts to increase use of the LNG-IUD and LARCs in Australia, particularly for young people, among whom rates of unplanned pregnancy and abortion are high [8, 9]. Local data indicate LARC use is associated with women’s increased age, geographical location and provider-level influences [6, 10–12]. Local data are often limited by small sample size, or aggregating LNG-IUDs with other LARCs/ IUDs. Designing and appropriately targeting interventions to increase LNG-IUD use requires knowledge of factors that may be systematically affecting its uptake at the population level. In the absence of reliable population-level measures of uptake, however, population-level prescription rates may be an appropriate substitute. Consequently, we analysed a national dataset of LNG-IUD prescriptions in Australia from 2008 to 2012. Our objectives were to calculate prescribing rates of the LNG-IUD in Australia, determine trends in uptake and associations with patient age, geographical location of the patient and proximity to specialist primary health services that might influence contraception prescription (family planning clinics and Aboriginal medical services).

## Methods

### Design & setting

This cross-sectional time-series analysis utilises a dataset of the number of prescriptions for the LNG-IUD through the PBS, obtained from the Department of Human Services for the maximum period available which ran from March 2008 to December 2012, inclusive. Prescriptions were recorded according to patient age (5-year cohorts) and residential location, as per standard statistical geography designations used by the Australian Bureau of Statistics. Given that Department of Human Service procedures meant that 2008 records were incomplete, whole-year prescriptions for 2008 were estimated by averaging the prescriptions written for the 10 months recorded and extrapolated across 12 months.

### Variables

Our outcome variable was the number of prescriptions for the LNG-IUD recorded in the PBS dataset.

Covariates included patient age, patient location (rural/regional/metro), presence of family planning clinics (FPC) at patient location, presence of an Aboriginal medical service (AMS) at patient location, and year of prescription. Patient age-group was measured in 5-year cohorts, from 15 to 49 years of age. Location was measured at Australian Statistical Geography Statistical Area Level 3 (SA3), statistical areas designed to reflect urban and regional areas sharing service centres or characteristics, with populations typically ranging from 30,000 to 130,000: these were assigned to remoteness categories per Australian Bureau of Statistics classifications [13]. The presence of FPCs within an SA3 was included as a binary variable (yes/no): only in WA did the FPCs [2] fall into the same SA3. The presence of Aboriginal medical services within an SA3 was recorded as a categorical variable indicating no service, a regional service, or remote service. Aboriginal medical services in remote areas of Australia are authorised to provide medication through a scheme outside the PBS. Prescription numbers of the LNG-IUD may appear artificially low in these areas, as they are not captured in our data. FPC locations were obtained from the Sexual Health & Family Planning Australia website [14]. The locations of Aboriginal medical services were identified via the National Aboriginal Community Controlled Health Organisation [15]. Prescription year was included as a linear term.

### Statistical analysis

LNG-IUD prescription rates were calculated, directly age standardised to the estimated resident population (hereafter ‘rates’) for each year using the population of women aged 15–49 years in Australia as at 30th June 2011 as the standard population [16]. Graphs were used to illustrate observed trends in LNG-IUD prescribing over time according to patient age, remoteness of patient location, and proximity to FPCs and Aboriginal medical services.

Poisson regression was used to examine associations between LNG-IUD prescribing and the covariates, adjusting for potential intra-cluster correlation at SA3 level. Prescriptions for which no SA3 were indicated (< 1.75% each year) were excluded from regression analyses. Analyses were stratified by age to investigate associations within the different age groups. Analyses were conducted using STATA Version 13 (College Station, TX: StataCorp LP).

## Results

### Summary of data and prescriptions

Absolute numbers of prescriptions rose from 60,522 in 2008 to 87,974 in 2012. Age-adjusted rates rose from 11.50 per 1000 women aged 15–49 (95% CI: 11.41, 11.59) in 2008 to 15.95 per 1000 women (95% CI: 15.95, 16.01; *p* < 0.01) in 2012 (Table 1). Total annual prescribing increased at approximately 9% per year (RR 1.09; 95% CI: 1.08, 1.09).

**Table 1:** Summary of available prescription data

Year	Total prescriptions	Prescriptions missing SA3 (% total)	Age-adjusted rate (per 1000 women)	95% CI
2008	60,522	1045 (1.73)	11.50	11.41, 11.59
2009	67,196	857 (1.28)	12.58	12.48, 12.67
2010	73,055	859 (1.18)	13.53	13.43, 13.63
2011	80,963	982 (1.21)	14.86	14.76, 14.96
2012	87,974	1045 (1.19)	15.95	15.85, 16.01

All figures relate to women aged 15–49

### Age

**Fig. 1:**
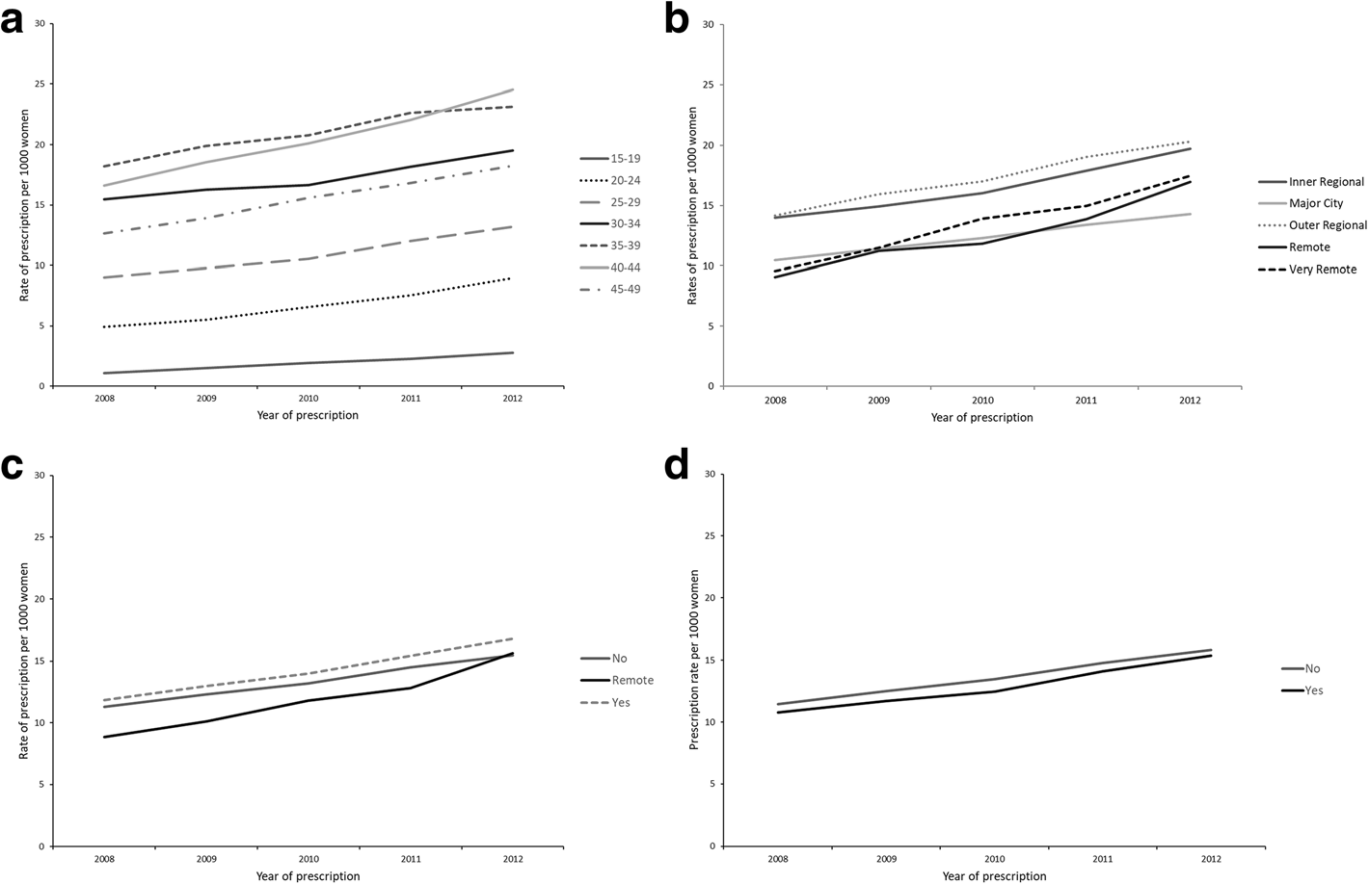
**a** Rates of prescription by age cohort. **b** Rates of prescription by residential location. **c** Rates of prescription by patient proximity to AMS. **d** Rates of prescription by patient proximity to Family Planning Clinics

Analysis of prescription data by age shows that prescribing has increased significantly across all age cohorts (*p* < 0.01). Rates were lowest in 15–19-year-olds, at 1.10 per 1000 in 2008 (95% CI, 0.97–1.24), increasing to 2.76 per 1000 women in 2012 (95% CI: 2.52–3.01). The absolute increase in prescribing rates was greatest for women 40–44 years old, rising from 16.73 per 1000 women in 2008 (95% CI: 16.12–17.34) to 23.77 per 1000 in 2012 (95% CI: 22.58–24.29) (Fig. 1a).

Stratified analyses indicate that rate of prescribing increased significantly each year for all age groups, being largest among 15–19-year-olds at 24% per year (IRR = 1.24; 95% CI: 1.22, 1.27) and among 20–24-year-olds at 16% per year (IRR = 1.16; 95% CI: 1.15, 1.18). (Table 2) Rate of prescribing increased least among 30–34-year-olds, at 5% per year (RR = 1.06; 95% CI:1.04, 1.06) (Table 2).

**Table 2:** Factors associated with IUD prescribing by age group

	15–19		20–24		25–29		30–34		35–39		40–44		45–49	
	IRR^1^	Adjusted IRR	IRR	Adjusted IRR	IRR	Adjusted IRR	IRR	Adjusted IRR	IRR	Adjusted IRR	IRR	Adjusted IRR	IRR	Adjusted IRR
Supply Year	1.24 (1.22–1.27)	1.24 (1.22–1.27)	1.16 (1.15–1.18)	1.16 (1.15–1.18)	1.09 (1.08–1.10)	1.10 (1.08–1.11)	1.05 (1.04–1.06)	1.05 (1.04–1.06)	1.06 (1.06–1.07)	1.06 (1.06–1.07)	1.09 (1.08–1.10)	1.09 (1.08–1.10)	1.10 (1.09–1.11)	1.10 (1.09–1.11)
Remoteness														
Major City	Ref.	Ref.	Ref.	Ref.	Ref.	Ref.	Ref.	Ref.	Ref.	Ref.	Ref.	Ref.	Ref.	Ref.
Inner Regional	1.62 (1.38–1.92)	1.57 (1.35–1.83)	2.09 (1.79–2.45)	2.08 (1.79–2.43)	1.99 (1.74–2.28)	2.01 (1.74–2.31)	1.52 (1.37–1.68)	1.54 (1.38–1.72)	1.21 (1.12–1.30)	1.22 (1.12–1.32)	1.09 (1.01–1.18)	1.10 (1.02–1.19)	1.07 (0.98–1.16)	1.07 (0.99–1.17)
Outer Regional	1.90 (1.59–2.26)	1.77 (1.48–2.13)	2.40 (2.01–2.87)	2.39 (1.97–2.89)	2.10 (1.81–2.42)	2.13 (1.80–2.52)	1.55 (1.39–1.72)	1.60 (1.40–1.83)	1.18 (1.09–1.29)	1.20 (1.09–1.33)	1.12 (1.04–1.21)	1.14 (1.05–1.24)	1.12 (1.03–1.21)	1.13 (1.03–1.24)
Remote	1.06 (0.58–1.93)	1.62 (1.00–2.63)	1.66 (0.95–2.93)	2.54 (1.78–3.63)	1.35 (0.80–2.30)	2.10 (1.47–3.00)	1.04 (0.68–1.59)	1.60 (1.25–2.07)	0.87 (0.61–1.26)	1.24 (0.92–1.67)	0.88 (0.62–1.24)	1.16 (0.83–1.62)	0.94 (0.76–1.17)	1.14 (0.87–1.50)
Very Remote	1.73 (1.06–2.83)	3.01 (1.98–4.58)	1.87 (1.28–2.75)	3.11 (2.06–4.68)	1.59 (1.16–2.19)	2.70 (1.83–3.93)	1.12 (0.91–1.39)	1.90 (1.36–2.64)	0.89 (0.74–1.07)	1.36 (0.93–1.98)	0.92 (0.69–1.21)	1.28 (0.86–1.91)	0.89 (0.68–1.16)	1.12 (0.74–1.70)
AMS														
None	Ref.	Ref.	Ref.	Ref.	Ref.	Ref.	Ref.	Ref.	Ref.	Ref.	Ref.	Ref.	Ref.	Ref.
Yes	1.40 (1.18–1.66)	1.16 (1.00–1.35)	1.33 (1.08–1.65)	1.02 (0.86–1.20)	1.20 (0.97–1.49)	0.96 (0.81–1.14)	1.07 (0.91–1.26)	0.93 (0.80–1.08)	1.03 (0.93–1.14)	0.97 (0.88–1.07)	1.01 (0.93–1.09)	0.97 (0.89–1.05)	1.01 (0.93–1.10)	0.98 (0.89–1.07)
Remote AMS	1.18 (0.69–2.02)	0.55 (0.32–0.95)	1.37 (0.91–2.05)	0.56 (0.36–0.89)	1.14 (0.80–1.61)	0.54 (0.35–0.83)	1.07 (0.68–0.02)	0.53 (0.38–0.75)	0.76 (0.62–0.94)	0.61 (0.42–0.88)	0.80 (0.62–1.05)	0.67 (0.45–1.00)	0.84 (0.69–1.03)	0.76 (0.53–1.10)
Family Planning														
No	Ref.	Ref.	Ref.	Ref.	Ref.	Ref.	Ref.	Ref.	Ref.	Ref.	Ref.	Ref.	Ref.	Ref.
Yes	1.21 (0.90–1.63)	1.25 (0.90–1.72)	1.09 (0.74–1.47)	0.97 (0.75–1.26)	0.95 (0.72–1.24)	0.97 (0.78–1.50)	0.92 (0.77–1.10)	0.93 (0.78–1.11)	0.96 (0.86–1.08)	0.97 (0.87–1.09	0.97 (0.87–1.07)	0.98 (0.88–1.08)	1.00 (0.89–1.12)	1.00 (0.89–1.13)

^1^: *IRR* incidence rate ratio

### Residential location

Rates increased significantly within all geographical locations (*p* < 0.01) (Fig. 1b) over time. Prescription rates were significantly higher among 15–34-year-olds not living in major cities (Table 2). Higher rates of prescription were observed among 15–19-year-old women living in very remote areas compared to major cities (RR: 3.01, 95% CI: 1.98–4.58), among 20–24-year-olds (RR: 3.11, 95% CI: 2.06–4.68) and among 25–29-year-olds (RR: 2.70, 95% CI: 1.83–3.93). Impact of remoteness decreased with increasing age. Among 35–39-year-olds and 40–44-year-olds, rates of prescribing increased in inner- and outer-regional areas – though to a lesser extent than among younger cohorts – with no significant increase seen in remote and very remote areas. Among 45–49-year-olds, rates were moderately higher in outer-regional areas than in major cities (RR 1.13, 95% CI: 1.03–1.24) (Table 2).

### Proximity to specialist health services

Although remote locations containing Aboriginal medical services had the lowest prescription rates across all years, these areas showed the greatest increase in number of prescriptions, rising from 8.86 per 1000 women in 2008 (95% CI: 7.47–10.24) to 15.63 per 1000 women in 2012 (95% CI: 13.31–17.95) (*p* < 0.01). Absolute rates were similar in locations with no AMS and less remote areas with an AMS (Fig. 1c) (*p* < 0.01). Prescriptions were significantly lower in remote areas with an AMS (RR: 0.62, 95% CI: 0.43–0.89). Women aged 15–39 years showed significantly lower rates of prescription in remote areas with an AMS, (Table 2).

Prescriptions rose at similar rates in locations with and without Family Planning clinics, the former increasing approximately 43% from 10.76 per 1000 women in 2008 (95% CI: 9.61–11.91) to 15.37 per 1000 women in 2012 (95% CI: 13.82–16.92) (*p* < 0.01), while in the latter prescriptions increased approximately 40% from 11.47 (95% CI: 11.08–11.86) to 15.82 per 1000 women in 2012 (95% CI: 15.34–16.31) (*p* < 0.01) (Fig. 1d). No significant associations were found between proximity to Family Planning clinics and prescription rates (Table 2).

## Discussion

### Prescription of LNG-IUD by age

Significant disparities exist in prescription of the LNG-IUD across age groups, with prescriptions markedly lower in younger cohorts. Health care providers can influence on women’s choice of contraceptive: lower prescriptions for young women may reflect persistent erroneous beliefs among providers about the suitability or otherwise of the LNG-IUD for young and nulliparous women [2, 17–20]. Alternatively, providers may recognise the suitability of LNG-IUD for nulliparous women, but believe its insertion is more complicated for this population, leading them to suggest other forms of contraception [20–22]. While LNG-IUD can be safely inserted in the outpatient setting, New Zealand data suggest that as many as a third of insertions may be undertaken under general anaesthetic, though reasons for doing so could not be assessed in the study [23]. It is possible that a newer, smaller form of the LNG-IUD, marketed internationally as Jaydess or Skyla may be easier to insert in younger/nulliparous women [24]. This device is not currently included on the PBS, nor been marketed locally.

That rates of prescription are increasing more quickly among younger women may reflect recent international attempts to educate providers regarding the safety of IUDs and importance of effective contraception for younger women. Some data also indicate that, having received evidenced-based information regarding contraceptive choices and offered the contraception of their choice free of charge, young women often prefer IUDs [25]. Ensuring that accurate information is available and accessible for young women may contribute to increased demand for, and subsequent prescription of, LARC.

With older women more likely to have had children than younger cohorts, higher rates of prescription of the LNG-IUD in this population may stem at least partly from the lack of these perceived barriers, or from increased demand for LARC from women who have completed their families. Evidence suggests that male and female sterilisations are declining, with the LNG-IUD a potentially contributing factor [4]. Prescription of LNG-IUD to older women may be higher given that the device may additionally be prescribed as a treatment for heavy menstrual bleeding, the incidence of which is associated with increased age [26]. Despite the likelihood of having had children, rates remain relatively low among the 30–34-year age group. While the LNG-IUD is appropriate for use post-partum, and is an effective method for timing pregnancies, prescription patterns in this age group may reflect a lack of knowledge among providers of this practice or barriers such as hospital policy [27].

### LNG-IUD prescription and residential location

We found differences in prescription patterns by patient location, with higher rates of prescription in regional areas compared to major cities. Prescription appears to be increasing in regional and remote areas. It may be that the particular circumstances of regional/rural living are increasing demand for LARC: difficulty in accessing health care, for example, may contribute to a desire for contraception requiring fewer points of contact with the health system. That this increased rate of prescription does not extend to remote areas, despite these populations facing many similar barriers, may indicate a lack of an appropriately trained workforce in remote areas. Analysis of current Family Planning training programs has shown that nurses as well as general practitioners can safely insert the LNG-IUD: this may be one avenue for addressing workforce shortages where necessary [28]. Even with increased accessibility of trained professionals, the relative ease of prescribing oral contraceptives may hinder prescription/insertion of the LNG-IUD – investigating means of incentivising primary care providers to do so may also facilitate uptake.

Prescription differs by geographic location across age groups. Women in regional and remote areas tend to have children at a younger age than their metropolitan counterparts [29]. This may increase the demand for the device in this population as these women have already completed their families. Providers may perceive these parous patients as more suitable candidates for the device, leading to increased prescription of the LNG-IUD. Limited data suggest that rates of hysterectomies, including as a potential treatment for menstrual disorders, may differ by age and urban/rural location: this may affect patterns of LNG-IUD prescription, though further investigation is necessary [30].

Lower rates of prescription were observed in areas with remote AMSs, potentially due to the capacity for some remote AMSs to prescribe outside the PBS system resulting in artificially low prescription numbers. It may also reflect a lack of an appropriately trained workforce in remote regions. Conversely, while no association was found between the presence of FPCs and prescription rates, despite the specialisation of FPCs in reproductive health, this may reflect the relatively small number and capacity of FPCs compared to the populations of regions they serve.

### Strengths and limitations

A major strength of this analysis is the use of PBS data, a national-level, administrative dataset which minimises selection and recall bias. However, the dataset has limitations. Data were available until 2012: there may have been a change in prescription patterns since this time, particularly given efforts in recent years to increase uptake. The number and location of FPCs may also have changed during the period analysed.

The data covers only PBS prescriptions, excluding those ineligible for PBS medications, and excludes prescriptions made through organisations which may provide the LNG-IUD outside the PBS through special dispensation regulations. This may affect our interpretation of findings regarding areas with remote AMSs, and potentially of metropolitan areas where prescriptions in hospitals may not be recorded. Whether lower rates of prescription in areas with remote AMSs found here reflect dispensation through these regulations or are indicative of broader barriers to access in remote areas, particularly for indigenous communities, warrants investigation. Examining patterns of prescriptions for the LNG-IUD in hospitals, and whether primary care providers in metropolitan regions are referring women to hospitals for prescription/insertion, may also be informative. While metropolitan prescriptions may appear artificially low in our data, however, the geographic pattern is likely to remain, with greater use of LARCs in rural areas having been identified elsewhere through women’s own reported use of contraception [12].

Finally, LNG-IUD may be dispensed through the PBS, but not inserted, meaning that our results reflect prescription of the device rather than uptake.

## Conclusions

Our analysis identified significant differences in patterns of LARC prescription, as a potential indicator of uptake, according to women’s age and area of residence. Further research in this area may include similar studies with more recent data, assessment of levels of awareness and accurate knowledge regarding the LNG-IUD in urban versus regional and remote populations at both patient and provider levels and comparisons of levels of provider comfort in LNG-IUD provision and insertion in urban versus rural locations. Identifying the services through which the device is prescribed – e.g. public versus private clinics, or primary care versus hospital-based clinics – may also help the interpretation of results presented here. The availability and accessibility of an appropriately trained workforce, in which settings they operate, and how this may differ across regions, also requires further investigation.

Understanding factors facilitating or constraining LNG-IUD uptake may have important implications for future sexual health and family planning policy, both in Australia and other countries with particularly low uptake of intrauterine contraceptives. Efforts aimed at continuing to increase levels of uptake among younger populations in particular should continue, with providers particularly encouraged to discuss the LNG-IUD with younger patients. However, particularly compared to other countries, there is scope for increased uptake of the LNG-IUD at all ages.

## Abbreviations

AMS: Aboriginal Medical Service
FPC: Family Planning Clinic
IUD: Intrauterine device
LNG-IUD: Levonorgestrel intrauterine device
PBS: Pharmaceutical Benefits Scheme
